# Bridging neurodevelopment and neurodegeneration in genetic frontotemporal dementia and Alzheimer's disease

**DOI:** 10.1002/alz70857_100515

**Published:** 2025-12-24

**Authors:** Youjin Jung, Jolina Lombardi, Rowan Heffelfinger, Sarah Inkelis, Christa Watson Pereira, Taru M. Flagan, Matthew Hall, Liya Rabkina, Zachary Miller, Stephan Sanders, Virginia E. Sturm, Jalayne J. Arias, Bruce L. Miller, Adam L. Boxer, John M Ringman, Maria Luisa Mandelli, Maria Luisa Gorno Tempini, Suzee E. Lee

**Affiliations:** ^1^ Memory and Aging Center, Department of Neurology, Weill Institute for Neurosciences, University of California, San Francisco, San Francisco, CA, USA; ^2^ Stanford University, Palo Alto, CA, USA; ^3^ University of Oxford, Oxford, United Kingdom; ^4^ School of Public Health ‐ Georgia State University, Atlanta, GA, USA; ^5^ Keck School of Medicine, University of Southern California, Los Angeles, CA, USA; ^6^ Department of Neurology, Memory and Aging Center, University of California San Francisco, San Francisco, CA, USA

## Abstract

**Background:**

The current conceptualization of genetic frontotemporal dementia (FTD) and Alzheimer's disease (AD) is that they are neurodegenerative diseases characterized by symptoms that develop late in life. Yet, studies in animal models and humans support the notion that autosomal dominant genes, whose variants cause these diseases, also play critical roles in neurodevelopment. Some studies have identified subtle behavioral, cognitive and neuroanatomical characteristics in asymptomatic adult genetic variant carriers. It remains unknown, however, how early in life these differences emerge and to what extent children and young adults with these variants may exhibit clinical overlap with neurodevelopmental disorders such as autism spectrum disorder (ASD), attention‐deficit hyperactivity disorder (ADHD), and language‐based learning disabilities (LBLD).

**Methods:**

At the UCSF Dyslexia Center, children and young adults (age range 7 to 25) from families with an autosomal dominant genetic variant for FTD or AD, participants with ASD, ADHD, LBLD, and typically developing children (TDC)/young adult controls, are being recruited for assessment. Our recruitment goals over the five‐year period include enrolling 50 participants from families with FTD, 50 participants from families with AD and 30 participants in each neurodevelopmental cohort. Measures collected include a neurological evaluation, neuropsychological testing, academic testing, and MRI brain scan (including T1, diffusion‐weighted imaging, resting‐state fMRI sequences). For participants from families with autosomal dominant FTD or AD, genetic testing for the variant in the family is performed on saliva specimens. Genetic variant status is not disclosed to participants or their families.

**Results:**

To date, we have evaluated 40 participants from families with FTD, seven from families with AD, two with ASD, 15 with ADHD, 33 with LBLD, and 18 TDCs with recruitment ongoing. Planned comparisons will examine the effects of these genetic variants on neuropsychological profiles, academic test scores, brain volume, white matter tract integrity and resting‐state functional connectivity networks.

**Conclusion:**

This study aims to characterize the neurodevelopmental phase of genetic FTD and AD, providing critical insights into their underlying biology and potential phenotypic overlap with neurodevelopmental syndromes.

